# Digital templating for the implantation of a curved short hip stem with an anterolateral MIS approach shows gender differences in digital templating

**DOI:** 10.1007/s00402-021-04005-9

**Published:** 2021-06-24

**Authors:** Matthias Luger, Rainer Hochgatterer, Matthias C. Klotz, Günter Hipmair, Tobias Gotterbarm, Bernhard Schauer

**Affiliations:** Department of Orthopaedics and Traumatology, Kepler University Hospital Linz, Krankenhausstrasse 9, 4020 Linz, Austria

**Keywords:** Digital templating, Short stem, Fitmore, Total hip arthroplasty, Minimally invasive, Anterolateral approach

## Abstract

**Purpose:**

Digital templating shows reliable accuracy for straight stem systems. In recent years, the implantation of short stems through minimally invasive approaches has gained more popularity. Minimally invasive approaches (MIS) show the risk of undersizing femoral components. Therefore, we questioned the planning adherence for a curved short stem and a bi-hemispherical acetabular cup implanted through an anterolateral MIS approach.

**Methods:**

A consecutive series of 964 hips (index surgery between 2014 and 2019) with Fitmore^®^ curved short stem and Allofit/-S^®^ acetabular cup (both ZimmerBiomet Inc, Warsaw, IN) were included. Preoperative digital templating was conducted anterior–posterior (AP) digital radiographs of the hip using mediCAD^®^ version 5.1 (Hectec GmbH, Altdorf, Germany). The templates of acetabular and femoral components (offset option and stem size) were retrospectively evaluated for general adherence, and according to sex, BMI and planner’s experience.

**Results:**

Planning adherence for the exact offset option was 70.6 and 21.6% for exact offset option and stem size. Adherence for acetabular cup  ± 1 size was 74.8%. A significant difference between male and female patients for the offset option could be found (*p* = 0.03, *z* = −2983). In 22.5% of male patients, an offset option one size higher and in 12.3% of female patients an offset option one size smaller than templated was used intraoperatively

**Conclusion:**

Digital templating for the Fitmore^®^ stem in cementless THA with a minimally invasive anterolateral approach shows comparable planning adherence to the existing literature for this cementless short stem. However, a lower planning adherence was detected compared to conventional straight stem systems. In male patients, the femoral offset is frequently undersized and in female patients frequently oversized compared to the preoperative plan. Surgeons should be aware of this difficulty in digital templating for Fitmore^®^ hip stem.

## Introduction

A successful postoperative outcome after total hip arthroplasty relies on restoring the biomechanics of the hip as well as selecting the appropriate implant size [1, 2]. Preoperative planning is seen as an integral part of selecting the correct implant size intraoperatively to avoid complications [3]. An underestimation of component sizes can lead to component loosening, while an overestimation can lead to intraoperative fractures [4–6]. The effective adherence of digital templating has been shown in several studies. The adherence for templating ranges from 78 to 98% within one size for the femoral stem, and between 80 and 91% to within 2 mm for the acetabular component [1, 7–10].

In recent years, cementless short stems have gained popularity [11]. Cementless short stems facilitate the use of minimally invasive approaches hence leading to less soft-tissue damage. In addition, cementless short stems reduce stress shielding and provide less bone loss [11, 12]. The choice of surgical approach was shown to have an effect on component size and position [1]. Rivera et al. showed an increased risk of underestimating the size of cementless short stems in total hip arthroplasty implanted through a direct anterior approach (DAA). An implantation of a femoral stem with a stem size at least two sizes smaller was six times higher in DAA without fluoroscopy compared to posterior approach (PA). Shemesh et al. [1] found an accuracy for stem size with an accuracy of 85% (to within one size) for the DAA and 77% for the PA (*p* = 0.71). Schmidutz et al. [13] found a comparable effective adherence for short stem arthroplasty with an average percentage of agreement (± 1 size) of 89.0% compared to 88.5% in conventional straight stem arthroplasty.

Digital templating for cementless short stems shows inconsistent findings for effective adherence in digital templating. Therefore, the purpose of this study was to evaluate the effective adherence of preoperative templating with cementless curved short stem and a bi-hemispherical acetabular cup in total hip arthroplasties using a minimally invasive anterolateral approach in supine position.

## Materials and methods

### Study design

In this retrospective study, a consecutive series of 987 THAs (930 patients, mean age 67.47 years ± 11.22) between 2014 and 2019 were screened for inclusion after approval of the local ethics committee (EK-No.: 1239/2019). All procedures performed in studies involving human participants were in accordance with the ethical standards of the institutional and/or national research committee and with the 1964 Helsinki declaration and its later amendments or comparable ethical standards.

THA was conducted using an anterolateral minimally invasive approach in supine position. Digital templating was carried out on the day before surgery by the surgeon or a fellow colleague using mediCAD^®^ version 5.1 (Hectec GmbH, Altdorf, Germany). These data were retrospectively analysed. Female and male patients with the age of 18–99 years were included. All included patients were operated for either severe, end-stage osteoarthritis or end-stage avascular necrosis of the femoral head (AVN) or mild hip dysplasia (Crowe I). Exclusion criteria were a history of prior surgery on the affected hip, THA for femoral neck fractures, post-traumatic osteoarthritis and complex deformities (i.e., severe hip dysplasia (Crowe  > 1), Legg–Calve–Perthes). Perioperative complications such as periprosthetic fractures led to exclusion of this study because of possible influence on component sizing. Nine hundred and sixty four hips in 907 patients met the inclusion criteria. 11 cases were excluded because of an intraoperative shaft fracture, 12 were excluded because of not meeting the right indication. Average age at operation was 67.47 years (SD ± 11.22 years). Gender distribution showed a higher predominance of female patients with 528 implantations in female patients (54.8%) to 436 implantations in male patients (45.2%). In 835 cases (86.6%), arthrosis was the indication, in 41 cases hip arthrosis due to hip dysplasia (4.3%) and in 88 cases avascular necrosis (9.1%). According to WHO criteria, 0.6% (*n* = 6) were underweight (BMI  < 18.5 kg/m^2^), 28.7% (*n* = 277) were in normal range (BMI  ≥ 18.5 and  < 25 kg/m^2^), 40.9% (*n* = 394) were overweight (BMI  ≥ 25 and  < 30 kg/m^2^), 20% (*n* = 193) were obese (BMI  ≥ 30 and  < 35 kg/m^2^), and 9.8% (*n* = 94) were severely obese (BMI  > 35 kg/m^2^).

All patients received an Allofit/-S^®^ acetabular cup and a Fitmore^®^ curved short stem (both Zimmer Inc., Warsaw, IN, USA). The Fitmore hip stem is an uncemented short curved hip stem available in four different offset options with a CCD-Angle ranging from 140 to 127° [A (140°), B (137°), B extended (129°) and C (127°)]. Every offset option is available in 14 different sizes. Allofit cup is a bi-hemispherical press fit cup with sizes ranging from 42 to 68 mm. The implantations were carried out by various surgeon with different level of experience including 11 consultants and seven residents. All consultants carry out more than 50 arthroplasties per year. Implantations by residents were done under the guidance of one of these experienced surgeons. In all cases, a minimally invasive anterolateral approach in supine position was used in a standardized manner using the interval between tensor fasciae latae and Gluteus medius.

### Preoperative X‑ray technique

A standardized preoperative digital radiograph anterior–posterior view of the hip was obtained in every case. Radiographs were taken with the patient in standing position and with both legs in 15° internal rotation. The beam was centered on the symphysis pubis. A standardized metallic radiopaque ball with a diameter of 25 mm was used as a reference for determining the magnification factor.

### Digital templating

Digital templating was carried out with mediCAD^®^ version 5.1 (Hectec GmbH, Altdorf, Germany) in a standardized manner. At first scaling and calculating the right magnification factor was done automatically by the software with the metallic radiopaque ball as the reference. Then, the center of rotation, the proximal femoral shaft axis and the leg length discrepancy were determined. After that the correct size for the acetabular component was determined. The acetabular component was placed at the floor of acetabulum, as this was the intended final position intraoperatively. Next the size of the femoral component was templated beginning with the correct offset option. The aim in templating a Fitmore curved short hip stem is to restore the anatomical offset by confirming that the medial curve of the stem follows closely the inner line of the cortex in the calcar region when the stem is in axis with the femoral canal. After choosing the correct stem family, the appropriate stem size is selected. The appropriate stem size is selected by choosing the stem which fills the intramedullary canal entirely. In general, surgeons tended to obey the predetermined offset option. In case of instability in trial reduction, they chose a higher offset option instead of the predetermined offset option.

### Statistical analysis

Statistical analysis was calculated with SPSS version 26 (IBM SPSS statistics, Chicago, IL, USA). The level of significance was *p* < 0.05. Descriptive analysis was done for the parameters age, sex, BMI and planner’s experience. A Shapiro–Wilk test was performed for testing for normality distribution. A Mann–Whitney *U* test was performed for testing the planning adherence in male and female patients. For testing of differences according to BMI and planner’s experience Kruskal–Willis test was carried out followed by a paired post-hoc test.

## Results

### General planning adherence

The exact offset option as templated was used in 70.6% (*n* = 681). In 16.5% of cases (*n* = 159) an offset option one size bigger than templated was used. In 1.8%, the used stem family was used with an offset option two times bigger. A smaller offset option one size smaller than templated was used in 10.7% (*n* = 103) and two sizes smaller in 0.4% (*n* = 4).

The exact offset option and stem size as templated was used in 21.6% (*n* = 208). A stem size within  ± 1 size was chosen in 30.2% cases (*n* = 291). In 14.4%, the stem size was within  ± 2 sizes, and in 4.5% (*n* = 43), the stem size was within  ± 3 sizes or more. In 29.4%, the stem size could not be evaluated because of the use of a different offset variant than templated.

The exact cup size as templated was used in 30.9% (*n* = 298). A cup size within  ± 1 size was used in 43.9% (*n* = 423), within  ± 2 sizes in 17.6% (*n* = 170), within  ± 3 sizes or higher in 7.6% (*n* = 73). The data for general reliability are shown in Table 1.

**Table 1:** General reliability for femoral and acetabular components

Offset option	Perfect match	− 1 offset option	+ 1 offset option	− 2 offset options	+ 2 offset options
	681 (70.6%)	159 (16.5%)	103 (10.7%)	4 (0.4%)	17 (1.8%)
Stem size	Perfect match	± 1 size	± 2 sizes	± 3 sizes and more	Offset option not correct
	208 (21.6%)	291 (30.2%)	139 (14.4%)	43 (4.5%)	283 (29.4)
Cup size	Perfect match	± 1 size	± 2 sizes	± 3 sizes and more	
	298 (30.9%)	423 (43.9%)	170 (17.6%)	73 (7.6%)	

### Sex and planning adherence

The same offset option as templated was used in 65.8% (*n* = 287) in male patients and in 74.6% in female patients (*n* = 394). In 22.5% of implantations (*n* = 98), in male patients, the used offset option was one size bigger than templated compared to 11.6% (*n* = 61) in female patients. In female patients, an offset option one size smaller than templated was implanted in 12.3% (*n* = 65) compared to 8.7% (38) in male patients.

Planning adherence for male and female patients showed a statistical significance for the offset option (*p* = 0.03, *z* = −2983). Table 2 shows the full results for templated adherence for the offset option according to gender.

**Table 2:** Adherence of digital templating according to gender

Offset option^1^	Perfect match	− 1 offset option	+ 1 offset option	− 2 offset options	+ 2 offset options
Male	287 (65.8%)	98 (22.5%)	38 (8.7%)	3 (0.7%)	10 (2.3%)
Female	394 (74.6%)	61 (11.6%)	65 (12.3%)	1 (0.2%)	7 (1.8%)

Stem size^2^	Perfect match	± 1 size	± 2 sizes	± 3 sizes and more	Offset option not correct
Male	77 (17.7%)	127 (29.1%)	65 (14.9%)	18 (4.1%)	149 (34.2%)
Female	131 (24.8%)	164 (31.1%)	74 (14.0%)	25 (4.7%)	134 (25.4%)

Cup^3^	Perfect match	± 1 size	± 2 sizes	± 3 sizes and more
Male	125 (28.7%)	189 (43.3%)	81 (18.6%)	41 (9.4%)
Female	173 (32.8%)	234 (44.3%)	89 (16.9%)	32 (6.1%)

^1^*p* = 0.03, *z* = −2983

^2^*p* = 0.683, *Z* = −0.409

^3^*p* = 0.072, *Z* = −1.802

The used femoral stem size was templated accurately in 17.7% (*n* = 77) in male patients and in 24.8% (*n* = 131) in female patients. Testing with Mann–Whitney *U* test shows no statistically significant difference for gender in templating the correct femoral stem size (*p* = 0.683, *Z* = −0.409). Table 2 shows the full results for femoral stem size according to gender.

The same cup size as templated was used in 28.7% (*n* = 125) in male patients and 32.8% (*n* = 173) in female patients. A cup size within  ± 1 size as templated was used in 43.3% (*n* = 189) in male patients and 44.3% (*n* = 234) in female patients. Mann–Whitney *U* test showed no statistically significant gender difference in templating the correct cup size (*p* = 0.072, *Z* = −1.802). Table 2 shows the full results for acetabular component size according to gender.

### BMI and planning adherence

The planning accuracy for the femoral shaft family and size is shown in Table 3. Testing with Kruskal–Willis test showed no statistically significant difference for the offset option (*p* = 0.825) and for the shaft size (*p* = 0.431) between the different BMI groups. Post hoc calculations showed no statistical significance. The planning accuracy for the acetabular component is shown in Table 3. Testing with Kruskal–Willis test showed no statistically significant difference for planning accuracy depending on BMI (*p* = 0.276). Post hoc calculations showed no statistical significance.

**Table 3:** Adherence of digital templating according to BMI

Offset option^1^	Perfect match	− 1 offset option	+ 1 offset option	− 2 offset options	+ 2 offset options
Underweight	4 (66.7%)	1 (16.7%)	1 (16.7%)	0 (0.0%)	0 (0.0%)
Normal weight	199 (71.8%)	38 (13.7%)	33 (11.9%)	6 (2.2%)	1 (0.4%)
Overweight	283 (71.8%)	56 (14.2%)	46 (11.7%)	6 (1.5%)	3 (0.8%)
Obese	131 (67.9%)	40 (20.7%)	19 (9.8%)	3 (1.6%)	0 (0.0%)
Severely obese	64 (68.1%)	24 (25.5%)	4 (4.3%)	2 (2.1%)	0 (0.0%)

Stem size^2^	Perfect match	± 1 size	± 2 sizes	± 3 sizes and more	Offset option not correct
Underweight	1 (16.7%)	1 (16.7%)	1 (16.7%)	1 (16.7%)	2 (33.3%)
Normal weight	67 (24.2%)	85 (30.7%)	48 (12.2%)	5 (1.8%)	78 (28.2%)
Overweight	86 (21.8%)	130 (33.0%)	48 (12.2%)	19 (4.8%)	111 (28.2%)
Obese	34 (17.6%)	51 (26.4%)	33 (17.1%)	13 (6.7%)	62 (32.1%)
Severely obese	20 (21.3%)	24 (25.5%)	15 (16.0%)	5 (5.3%)	30 (31.9%)

Cup size^3^	Perfect match	± 1 size	± 2 sizes	± 3 sizes and more
Underweight	1 (16.7%)	5 (83.3%)	0 (0.0%)	0 (0.0%)
Normal weight	91 (32.9%)	118 (42.6%)	46 (16.6%)	22 (7.9%)
Overweight	106 (26.9%)	186 (47.2%)	67 (17.0%)	35 (8.9%)
Obese	61 (31.6%)	77 (39.9%)	43 (22.3%)	12 (6.2%)
Severely obese	39 (41.5%)	37 (39.4%)	14 (14.9%)	4 (4.3%)

^1^p = 0.825

^2^p = 0.431

^3^p = 0.276

### Planner’s experience

The surgeons were divided according to their experience in implantation of the specific short curved hip stem into three different groups. Group 1 consisted of surgeons with experience in more than 100 implantations of short stem hip arthroplasty through a minimally invasive anterolateral approach, group 2 consisted of surgeons with experience between 50 and 100 implantations and group 3 consisted of surgeons with less than 50 implantations. Testing with Kruskal–Willis test showed no statistically significant difference for planning adherence depending on planner’s experience for offset option, stem size and cup size (*p* = 0.298, *p* = 0.074, *p* = 0.076) Post hoc calculations showed no statistically significance. The planning accuracy depending on planner’s experience is shown in Table 4.

**Table 4:** Adherence of digital templating according to planner’s experience

Offset option^1^	Perfect match	− 1 offset option	+ 1 offset option	− 2 offset options	+ 2 offset options
Group 1	373 (70.1%)	92 (17.3%)	55 (10.3%)	11 (2.1%)	1 (0.2%)
Group 2	241 (69.7%)	56 (16.2%)	43 (12.4%)	4 (1.2%)	2 (0.6%)
Group 3	67 (77.9%)	11 (12.8%)	5 (5.8%)	2 (2.3%)	1 (1.2%)

Stem size^2^	Perfect match	± 1 size	± 2 sizes	± 3 sizes and more	Offset option not correct
Group 1	121 (22.7%)	141 (26.5%)	85 (16.0%)	26 (4.9%)	159 (29.9%)
Group 2	70 (20.2%)	119 (34.4%)	41 (11.8%)	11 (3.2%)	105 (30.3%)
Group 3	17 (19.8%)	31 (36.0%)	13 (15.1%)	6 (7.0%)	19 (22.1%)

Cup^3^	Perfect match	± 1 size	± 2 sizes	± 3 sizes and more
Group 1	163 (30.6%)	220 (41.4%)	93 (17.5%)	56 (10.5%)
Group 2	110 (31.8%)	159 (46.0%)	61 (17.6%)	16 (4.6%)
Group 3	25 (29.1%)	44 (51.2%)	16 (18.6%)	1 (1.2%)

^1^*p* = 0.298

^2^*p* = 0.074

^3^*p* = 0.076

## Discussion

We retrospectively analyzed the planning adherence for a short curved hip stem and a bi-hemispherical acetabular cup in 964 implantations through minimally invasive anterolateral approach in supine position. The existing data regarding accuracy of preoperative digital templating focuses mainly on the planning adherence in hip arthroplasty with straight stem systems. Holzer et al. [6] found a planning accuracy of 42% for predicting the exact size and 87% for templating within a range of  ± 1 size for straight stem systems. In this study, a similar planning adherence for the same short stem could not be found with a correctly predicted offset option and stem size in only 21.6. In 30.2%, the templated stem size was implanted within the range of  ± 1 stem size. However, more offset options are available for implanting the curved short stem reviewed in this study. Planning adherence depends on an initially correct choice of the offset option. Rivera et al. [14] compared the used femoral component size in direct anterior approach (DAA) and posterior approach (PA) with the same curved short hip stem. A non-agreement between predicted size in acetate templating and implanted femoral component was found in 42.37% for PA and 66.04% in DAA. Non-agreement was defined with any size discrepancy. Rivera et al. [14] did not analyze the adherence for the offset option and the stem size separately. In addition, it was not defined in the study if a falsely predicted offset option automatically counted as a non-agreement. Therefore, comparison with our findings is not fully possible.

Jung et al. [15] conducted a study on the validity of digital templating and the impact of stem design and planner’s experience. They found a planning adherence for the same curved short stem with 34.4, 21.9 and 12.5% for three differently experienced planning surgeons. The planning adherence of predicting the correct offset option was 65.6, 62.5 and 25% depending on the planner’s experience. Compared to our study, we found a similar general planning adherence with 70.6% for offset option and 21.6% for exact offset option and stem size. A statistically significant difference in planning adherence according to planner’s experience could not be found in our results. However, planner’s experience was defined differently in our study compared to Jung et al. [15]. In our study, we included surgeons with high experience in total hip arthroplasty with straight stem systems and with low experience in short stem arthroplasty with in short stem hip arthroplasty and vice versa. Therefore, we defined planner’s experience according to the number of implantations of curved short stems. In addition, we postulate that our results do not show a difference according to surgeon’s experience, because in all groups, we have different levels of planning adherence regardless of experience. On average, all groups show a similar planning adherence, because in all groups, we included surgeons, who follow their preoperative template in most cases, while other disobey more frequently.

The planning adherence for templating the correct offset option goes up to 91.4% in other studies for femoral stem system which offer two different offset options [16]. The planning adherence for straight stems with three different offset options ranges between 67.9 and 42.9% [15]. In contrast, the used short stem in this study offers four different offset option. Our findings with an adherence of 70.6% for templating the correct offset option show comparable results to other findings with stem systems with less offset options. In addition, these studies show a lower number of cases between 60 and 112 implantations compared to 964 implantations in this study [14–16].

The planning adherence for the bi-hemispherical acetabular cup shows similar results to other studies. We found a planning adherence of 30.9% of predicting the cup size and 43.9% in a range of  ± 1 size. The planning adherence for the bi-hemispherical acetabular cup was found to be between 35 and 26.8% depending on the planner’s experience [15]. Our findings show a comparable planning adherence for the implantation of this acetabular component in minimally invasive supine anterolateral hip arthroplasty depending on the planner’s experience with an adherence of 30.9% in the all patients and between 30.6 and 29.1%.

Statistical analysis found a significant sex difference in templating the correct offset option of the curved short stem in this study (*p* = 0.003). In male patients, an offset option one size higher than templated was used in 22.5% compared to 11.6% in female patients. In female patients, an offset option one sizer smaller than templated was implanted in 12.3% compared to 8.7% in male patients. These findings show the potential of underestimating hip offset in digital templating for male patients and overestimating hip offset in female patients. Merle et al. [17] demonstrated that femoral offset is underestimated by 13% on AP radiographs of the pelvis when compared with CT. This could be major factor for the under/oversizing of offset option in digital templating of Fitmore^®^ hip stem. In male patients, the femoral offset is higher on average. In combination with underestimating femoral offset on AP X-ray of the pelvis, this could lead to a relative increase in underestimation of femoral offset and, therefore, also underestimation of the offset option in digital templating. Reconstruction of the hip offset is essential for the postoperative outcome [18]. Especially, the used curved short stem with its four different offset options is designed to accomplish a better reconstruction of hip offset. The reduction of acetabular offset in total hip arthroplasty is usually compensated in increasing the femoral offset. These changes in femoral and acetabular offset could pose as a possible source of error in digital templating of a curved short stem with four different offset options leading in under or overestimation of the templated offset option in the reconstruction of hip offset. Warnock et al. [19] described a higher loss of acetabular offset, while reaming the acetabulum in male patients due to greater acetabular floor depth and higher neck cuts leading to more lengthening in female patients. These findings could also be a factor for the results in our study. The more frequent use of a lower offset option than templated in female patients was probably chosen because of a lower reduction of acetabular offset and higher stability due to leg lengthening. A statistically significant difference in stem size and cup size could not be found in our study (*p* = 0.683, *p* = 0.072) comparable to findings in other studies [6].

The influence of BMI on the adherence of templating showed no statistically significant difference. Sershon et al. [19] also did not find a statistically significant impact of BMI on the planning adherence of femoral and acetabular components in digital templating in a study with 603 patients. Other studies showed a statistically significant difference for normal weight and overweight patients, but not for obese or severely obese patients [6]. A mispositioning of the radiopaque reference could pose as a magnification error for digital templating in obese patients [20] but was not confirmed statistically in other studies [21].

Limitations of this study are the retrospective design and the different experience in templating of different surgeons. In some cases, experienced surgeons in arthroplasty have been beginners in templating and implantation of curved short stems, while in some cases, residents without any experience carried out the templating for fellow surgeons. Another limitation of this study is the solely usage of AP radiographs of the pelvis. Additional use of AP radiographs of the affected hip could have improved planning adherence.

## Conclusion

Digital templating for the Fitmore^®^ stem in cementless THA with a minimally invasive anterolateral approach shows comparable planning adherence to the existing literature for this cementless short stem. However, a lower planning adherence was detected compared to conventional straight stem systems. In male patients, the femoral offset is frequently undersized and in female patients frequently oversized compared to the preoperative plan. Surgeons should be aware of this difficulty in digital templating for Fitmore^®^ hip stem.

## References

[1] Shemesh SS, Robinson J, Keswani A, Bronson MJ, Moucha CS, Chen D (2017). The accuracy of digital templating for primary total hip arthroplasty: is there a difference between direct anterior and posterior approaches?. J Arthroplasty.

[2] Bono JV (2004). Digital templating in total hip arthroplasty. J Bone Joint Surg Am.

[3] Eggli S, Pisan M, Muller ME (1998). The value of preoperative planning for total hip arthroplasty. J Bone Joint Surg Br.

[4] Heinert G, Hendricks J, Loeffler MD (2009). Digital templating in hip replacement with and without radiological markers. J Bone Joint Surg Br.

[5] Kim YH, Kim VE (1993). Uncemented porous-coated anatomic total hip replacement. Results at six years in a consecutive series. J Bone Joint Surg Br.

[6] Holzer LA, Scholler G, Wagner S, Friesenbichler J, Maurer-Ertl W, Leithner A (2019). The accuracy of digital templating in uncemented total hip arthroplasty. Arch Orthop Trauma Surg.

[7] Gamble P, de Beer J, Petruccelli D, Winemaker M (2010). The accuracy of digital templating in uncemented total hip arthroplasty. J Arthroplasty.

[8] Kumar PG, Kirmani SJ, Humberg H, Kavarthapu V, Li P (2009). Reproducibility and accuracy of templating uncemented THA with digital radiographic and digital TraumaCad templating software. Orthopedics.

[9] Shaarani SR, McHugh G, Collins DA (2013). Accuracy of digital preoperative templating in 100 consecutive uncemented total hip arthroplasties: a single surgeon series. J Arthroplasty.

[10] Steinberg EL, Shasha N, Menahem A, Dekel S (2010). Preoperative planning of total hip replacement using the TraumaCad system. Arch Orthop Trauma Surg.

[11] Gkagkalis G, Goetti P, Mai S, Meinecke I, Helmy N, Bosson D, Kutzner KP (2019). Cementless short-stem total hip arthroplasty in the elderly patient—is it a safe option?: a prospective multicentre observational study. BMC Geriatr.

[12] Gustke K (2012). Short stems for total hip arthroplasty: initial experience with the Fitmore stem. J Bone Joint Surg Br.

[13] Schmidutz F, Steinbruck A, Wanke-Jellinek L, Pietschmann M, Jansson V, Fottner A (2012). The accuracy of digital templating: a comparison of short-stem total hip arthroplasty and conventional total hip arthroplasty. Int Orthop.

[14] Rivera F, Leonardi F, Evangelista A, Pierannunzii L (2016). Risk of stem undersizing with direct anterior approach for total hip arthroplasty. Hip Int.

[15] Jung S, Neuerburg C, Kappe T, Wernerus D, Reichel H, Bieger R (2012). Validity of digital templating in total hip arthroplasty: impact of stem design and planner's experience. Z Orthop Unfall.

[16] Unnanuntana A, Wagner D, Goodman SB (2009). The accuracy of preoperative templating in cementless total hip arthroplasty. J Arthroplasty.

[17] Merle C, Waldstein W, Pegg E, Streit MR, Gotterbarm T, Aldinger PR, Murray DW, Gill HS (2012). Femoral offset is underestimated on anteroposterior radiographs of the pelvis but accurately assessed on anteroposterior radiographs of the hip. J Bone Joint Surg Br.

[18] Kutzner KP, Kovacevic MP, Roeder C, Rehbein P, Pfeil J (2015). Reconstruction of femoro-acetabular offsets using a short-stem. Int Orthop.

[19] Sershon RA, Diaz A, Bohl DD, Levine BR (2017). Effect of body mass index on digital templating for total hip arthroplasty. J Arthroplasty.

[20] Gonzalez Della Valle A, Comba F, Taveras N, Salvati EA (2008). The utility and precision of analogue and digital preoperative planning for total hip arthroplasty. Int Orthop.

[21] Heep H, Xu J, Lochteken C, Wedemeyer C (2012). A simple and convenient method guide to determine the magnification of digital X-rays for preoperative planning in total hip arthroplasty. Orthop Rev (Pavia).

